# Supplementary material for: Detecting chromatin state alterations in PBMCs associated with Type 2 Diabetes Mellitus
Source: Commun Med (Lond). 2026 Mar 17;6:268. doi: 10.1038/s43856-026-01513-w (PMC13144323; doi:10.1038/s43856-026-01513-w)
Supplement: Supplementary file 2 — Supplemental Information [file 43856_2026_1513_MOESM2_ESM.pdf]

Supplementary Material for

Detecting chromatin state alterations in PBMCs associated with Type 2 Diabetes Mellitus

Maryam Moazeni Afarani[1,†], Rajshikhar Gupta[1,2,†],
Caroline Uhler[3,4], Issa Fetian[5,6], GV Shivashankar[1,2*]
[1]Laboratory of Multiscale Bioimaging, Paul Scherrer Institut, Villigen, Aargau, Switzerland,
[2]Department of Health Sciences and Technology, ETH Zürich, Zürich, Switzerland,
[3]Laboratory of Information and Decision Systems, MIT, Cambridge, USA
[4]Eric and Wendy Schmidt Center, Broad Institute of MIT and Harvard, Cambridge, USA
[5]Hausarztpraxis MZ Brugg, Switzerland
[6]Universität Basel, Basel, Switzerland
*To whom correspondence should be addressed; E-mail: gshivasha@ethz.ch
† These authors contributed equally and are co-first authors.

Descriptive handcrafted chromatin feature list

We present the list of features for the multi-parametric analysis of the chromatin organization across different stages of T2DM progression. We have divided these features into three categories to describe the 3D spatial chromatin distribution, organization, and 2D/3D morphology of the nucleus.

- 2D Morphometrics: Projection of segmented nuclear volume
  1. Aspect ratio, area, bounding box height, bounding box width, maximum and minimum caliper distance, roundness, shape factor, circularity, concavity of 2D Projection of nuclear volume.
  2. Mean and standard deviation of centroid to edge distance of 2D projection of nuclear volume.
  3. Semi-major axis length of the fitted ellipse, Semi-minor axis length of the fitted ellipse to the 2D projection of nuclear volume.
- 3D Morphometrics: nuclear volume
  1. $R1$, $R2$, $R3$ semi-axes length of ellipsoid fit to 3D nuclear volume.
  2. Volume, compactness, elongation, ferret distance and Flatness of 3D nuclear volume.
  3. Maximum, mean, minimum, and standard deviation of 3D nuclear surface to centroid distance.
  4. Sparseness, sphericity, surface area of nuclear volume.
  5. Volume fraction of nuclear volume to bounding cuboid and ellipsoid.
  6. Volume of bounding ellipsoid to nuclear volume.
- Spatial intensity distribution
  1. Mean, median, mode and standard deviation of range normalized intensity.
  2. Variance, Skewness, Entropy, Kurtosis, Standard deviation, Volume normalization of values of intensity.

3. Distance between geometric vs intensity weighted centroid, Ratio of number voxels with Highest intensity to Lowest intensity, Ratio of a number of voxels with an intensity value greater than 80 percentile to less than 20 percentile.

- Heterochromatin domain organization

1. Number of heterochromatin domains, Ratio of heterochromatin domain volume, and total nuclear volume.
2. Maximum, mean, median, minimum, and standard deviation of
   (a) $D1$: inter heterochromatin domain centroid to centroid distance,
   (b) $D2$: inter heterochromatin domain surface to surface distance.
3. Maximum, mean, median, minimum, and standard deviation, and a total of
   (a) $D3$: heterochromatin domain centroid to nuclear centroid distance,
   (b) $D4$: the nearest distance of heterochromatin domain surface to nuclear centroid,
   (c) $D5$: the nearest distance of heterochromatin domain surface to nuclear surface,
   (d) $D6$: the radial distance between heterochromatin domain surface to nuclear surface,
   (e) $F1$: the ratio of $D3$ to radial distance R,
   (f) $F2$: the ratio of $D6$ to radial distance R,
   (g) heterochromatin domain volume.

Sample No	Date of collection	Sex	Age-Group	Primary diagnosis	Secondary diagnosis	Tertiary diagnosis
2	6/29/2023	F	26-30	Healthy	-	-
6	7/27/2023	M	36-40	Prediabetes	Hepatitis B	-
12	8/2/2023	M	71-75	Diabetes	Hypertension	Chronic Venous Insufficiency
15	8/8/2023	M	61-65	Prediabetes	Fatty liver	Pancreatic Lipomatosis
22	8/17/2023	F	26-30	Prediabetes	-	-
23	8/18/2023	M	46-50	Diabetes	Hypertension	-
26	8/18/2023	F	51-55	Prediabetes	Hypercholesterolemia	-
30	8/22/2023	F	61-65	Prediabetes	Morbus Crohn	-
35	8/23/2023	M	51-55	Diabetes	Hypertension	-
37	8/23/2023	M	61-65	Prediabetes	Hypertension	-
39	8/23/2023	F	26-30	Healthy	-	-
40	8/24/2023	F	56-60	Prediabetes	Hyperlipidemia	-
41	8/24/2023	F	61-65	Prediabetes	Hyperlipidemia	-
43	8/29/2023	M	36-40	Prediabetes	-	-
44	9/1/2023	M	41-45	Healthy	-	-
45	9/5/2023	M	51-55	Prediabetes	Hypertension	-
46	9/5/2023	M	41-45	Diabetes	Hypertension	-
47	9/11/2023	M	61-65	Prediabetes	Hypertension	Hyperlipidemia
48	9/12/2023	M	41-45	Prediabetes	-	-
49	9/12/2023	F	61-65	Diabetes	Hypertension	Hyperlipidemia
51	9/12/2023	M	66-70	Prediabetes	Hypertension	-
52	9/14/2023	M	56-60	Prediabetes	Hypertension	Hyperlipidemia
54	9/19/2023	M	71-75	Diabetes	Hypertension	Hyperlipidemia
55	9/19/2023	M	61-65	Diabetes	Hypertension	Hyperlipidemia
60	9/20/2023	M	66-70	Diabetes	Hypertension	Hyperlipidemia
61	9/21/2023	F	66-70	Diabetes	Hypertension	Hyperlipidemia
62	9/21/2023	F	61-65	Diabetes	Hyperlipidemia	-
63	9/21/2023	F	66-70	Prediabetes	Hypertension	Hyperlipidemia
64	9/21/2023	M	56-60	Diabetes	Hypertension	Hyperlipidemia
65	9/22/2023	M	61-65	Diabetes	Hypertension	Hyperlipidemia
66	9/22/2023	M	61-65	Prediabetes	-	-
67	9/22/2023	F	66-70	Diabetes	Hyperlipidemia	-
69	9/25/2023	F	51-55	Diabetes	Hyperlipidemia	-
73	9/28/2023	M	66-70	Prediabetes	Hypertension	Hyperlipidemia
74	9/28/2023	M	56-60	Prediabetes	Hyperlipidemia	-
76	9/28/2023	M	71-75	Diabetes	Hypertension	-
78	10/2/2023	M	76-80	Prediabetes	Hypertension	Hyperlipidemia
80	10/3/2023	F	71-75	Diabetes	Hypertension	Hyperlipidemia
82	10/3/2023	M	61-65	Prediabetes	Hyperlipidemia	-
85	10/5/2023	M	66-70	Prediabetes	Hypertension	Hyperlipidemia
86	10/12/2023	F	66-70	Diabetes	Hypertension	Hyperlipidemia
91	10/23/2023	F	46-50	Prediabetes	-	-
95	11/24/2023	M	21-25	Healthy	-	-
96	11/24/2023	F	31-35	Healthy	-	-
97	11/24/2023	F	21-25	Healthy	-	-
98	11/28/2023	F	26-30	Healthy	-	-
99	11/28/2023	M	36-40	Healthy	-	-
103	1/15/2024	M	41-45	Healthy	-	-
105	3/5/2024	F	51-55	Healthy	-	-
106	3/5/2024	M	56-60	Healthy	Thalassemia minor	-
107	3/14/2024	F	41-45	Healthy	Seasonal allergy	-
108	3/14/2024	F	31-35	Healthy	Fibroadenoma mama left	-
109	4/4/2024	M	41-45	Healthy	-	-
110	4/5/2024	F	46-50	Healthy	-	-
111	4/24/2024	F	41-45	Healthy	Hypercholesterolemia	-
112	4/26/2024	M	41-45	Healthy	-	-
113	5/1/2024	M	46-50	Healthy	-	-
114	5/10/2024	F	36-40	Healthy	-	-
115	5/16/2024	F	41-45	Healthy	-	-
116	5/17/2024	F	46-50	Healthy	-	-
117	5/17/2024	M	41-45	Healthy	-	-
118	5/24/2024	M	31-35	Healthy	-	-

TABLE S1: Clinical description of the human blood samples presented exactly as received from the Hausarztpraxis MZ Brugg, Switzerland, focusing on key demographic and diagnostic information pertinent to this study.

Disease Stage	Cell Counts (Total)	Cell Counts (Sampled)
Diabetic	8124	3317
Healthy	8945	3303
Prediabetic	7145	3316

TABLE S2: Total number of live PBMC nuclei imaged for every disease stage before and after down-sampling.

Dimension	HbA1c	CRP / IL-6	Chromatin imaging (this work)
Primary signal	Long-term glycemia (2-3 months) [1], non indicative of patient specific inflammation	Systemic inflammation [2]	Alteration in chromatin organization in PBMCs due to sub-population stratification, activation or senescence
Clinical role	Diagnostic & monitoring standard	Adjunct risk / inflammation marker	Exploratory/adjunct; Orthogonal to established methods
Diagnostic power	Established cut-off: $HbA_1c \geq 6.5\%$; high specificity($\geq$ 90% [3]); Moderate sensitivity (45.5%[3],70%[4])	Associated with risk but non-specific [5, 2] CRP: 1.5–12.0 (rel. risk = 4.2); IL-6 Range: 0.9–5.6 (rel. risk = 2.3) [5], multivariate accuracy 76-78% [2]	specificity of 0.78 (CI: 0.4 - 0.972) and sensitivity of 0.75 (CI: 0.194 - 0.994) in distinguishing diabetic individuals within cohort.
Cost per test (relative)	Low ($8 – $16) [6]	CRP: Low ($12 – $16)[7]; IL-6: Moderate: ($100 - $300) [8]	Low (Estimated with Scalability): $\leq$ $10; includes low cost reagents like Hoechst ($143/10 mL Thermo Fischer) & Microfluidics Devices [9]
Capital/equipment	Standard clinical analyzer	Standard immunoassay platforms	Benchtop centrifuge + density-gradient separation + confocal microscope (or high-NA wide-field) + compute for ML
Ease of use	High	High	automation feasible
Throughput / TAT	High / same-day	High / hours	Moderate (same-day feasible); automation can increase throughput
Scalability	Mature	Mature	Scalable with plate-based imaging, automated segmentation, model deployment; not POC yet
Complementarity	Glycemic exposure	Inflammatory burden	Promising sensitive and specific approach in the context of this cohort size; orthogonal to established methods.

TABLE S3: Comparison of HbA1c, CRP/IL-6, and chromatin imaging assay across diagnostic and practical dimensions.

Class	Th	Sens	Sens CI Low	Sens CI High	Spec	Spec CI Low	Spec CI High	ROC AUC OvR	PR AUC OvR
Diabetic	0.293	0.75	0.194	0.994	0.778	0.400	0.972	0.833	0.710
Prediabetic	0.577	0.75	0.194	0.994	0.556	0.212	0.863	0.694	0.586
Healthy	0.433	0.20	0.005	0.716	0.875	0.473	0.997	0.575	0.469

TABLE S4: Per-class performance metrics with thresholds, sensitivity/specificity (with CIs), and OvR AUCs. for independent test set. Target sensitivity for Diabetes is 0.8, Prediabetes is 0.8, and the target Specificity for Healthy is 0.80.

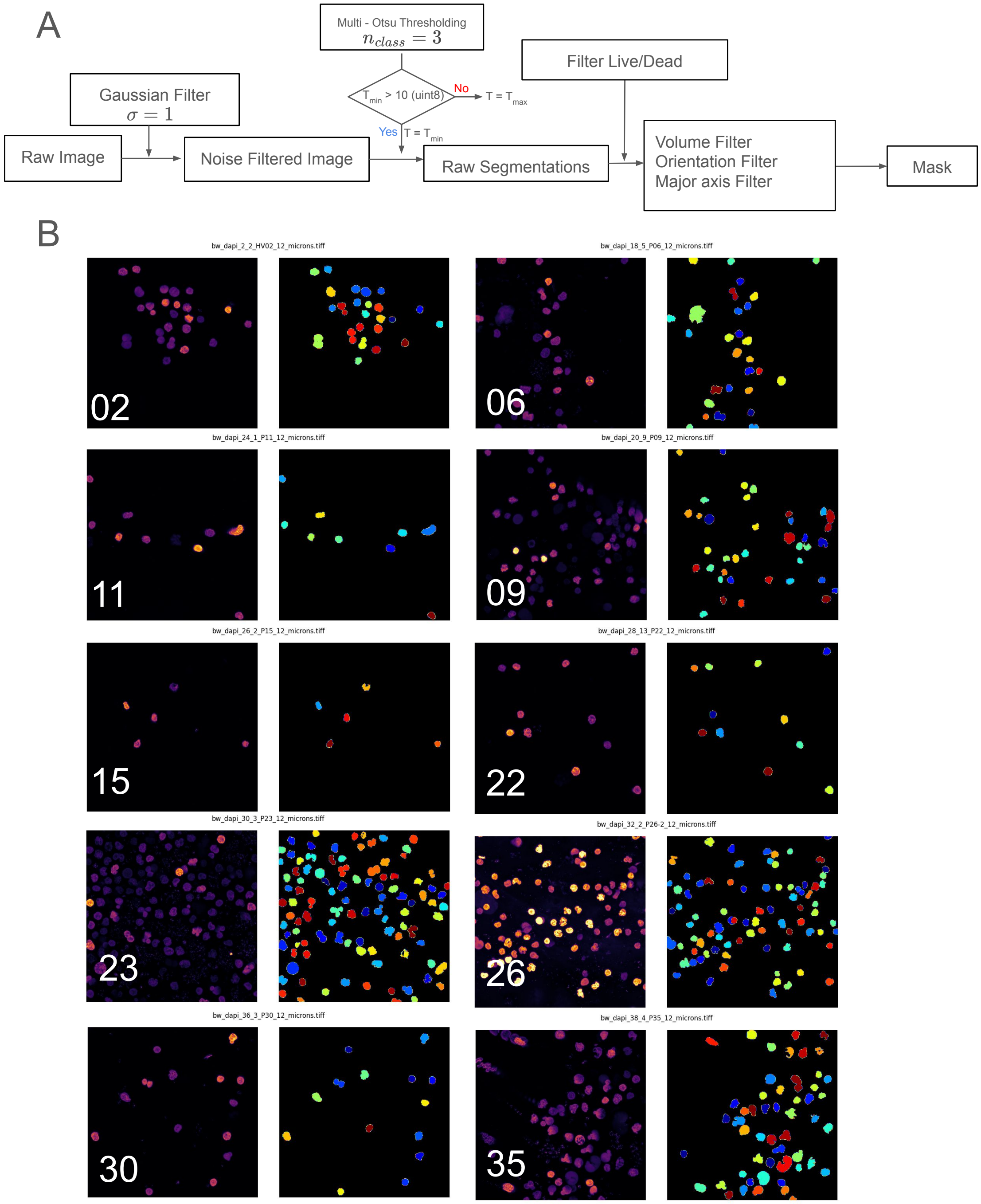

FIG. S1: (A) Sample No. 2 to 35: Representative maximum z projected confocal images stained with Hoechst (left) and corresponding nuclear segmentations (right). The segmentation is colormapped (colormap: jet) and randomly shuffled to enhance visual distinction and clarity. The number annotations to images represent the corresponding Sample No (see Table 1.)

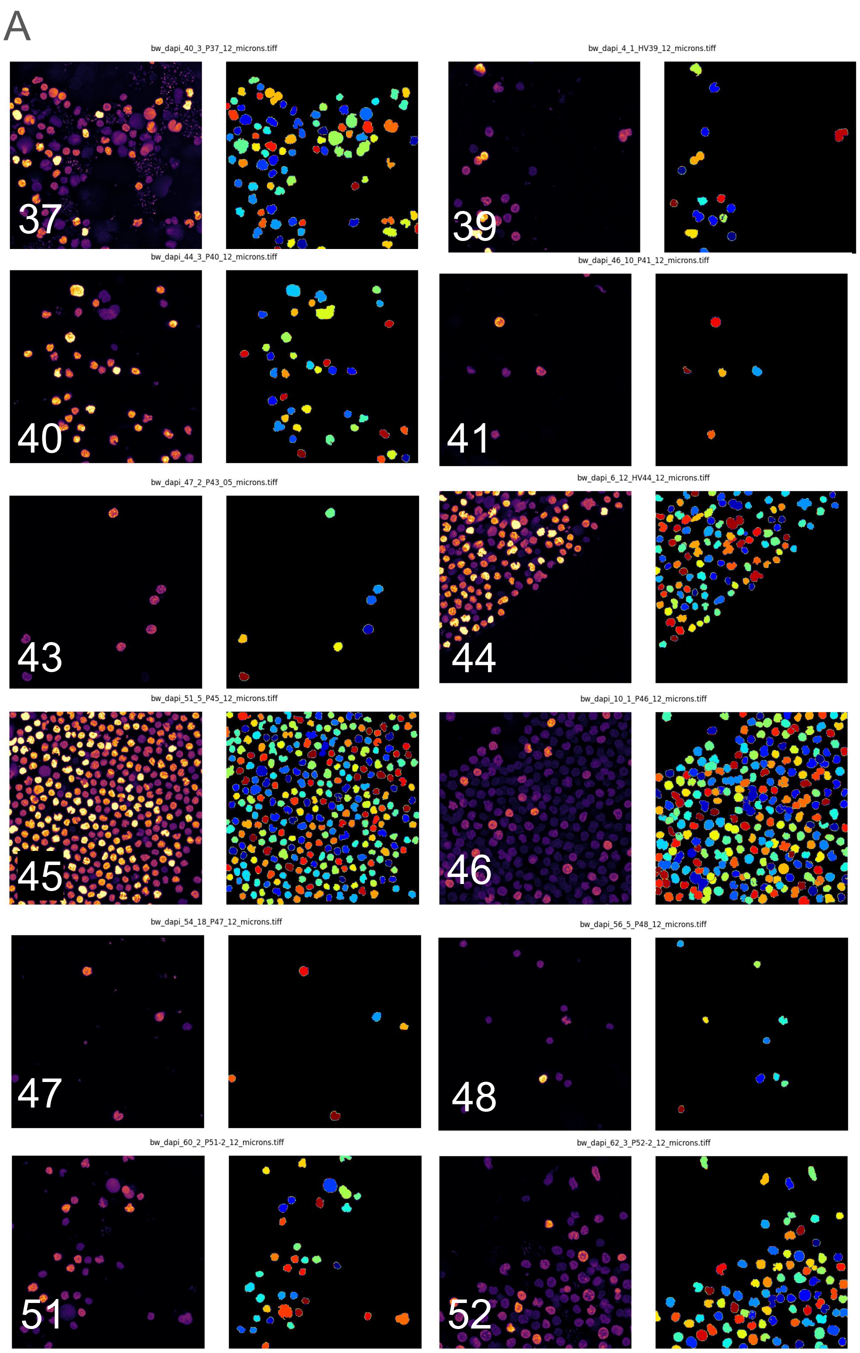

FIG. S2: (A) Sample No. 37 to 52: Representative maximum z projected confocal images stained with Hoechst (left) and corresponding nuclear segmentations (right). The segmentation is colormapped (colormap: jet) and randomly shuffled to enhance visual distinction and clarity. The number annotations to images represent the corresponding Sample No (see Table 1.)

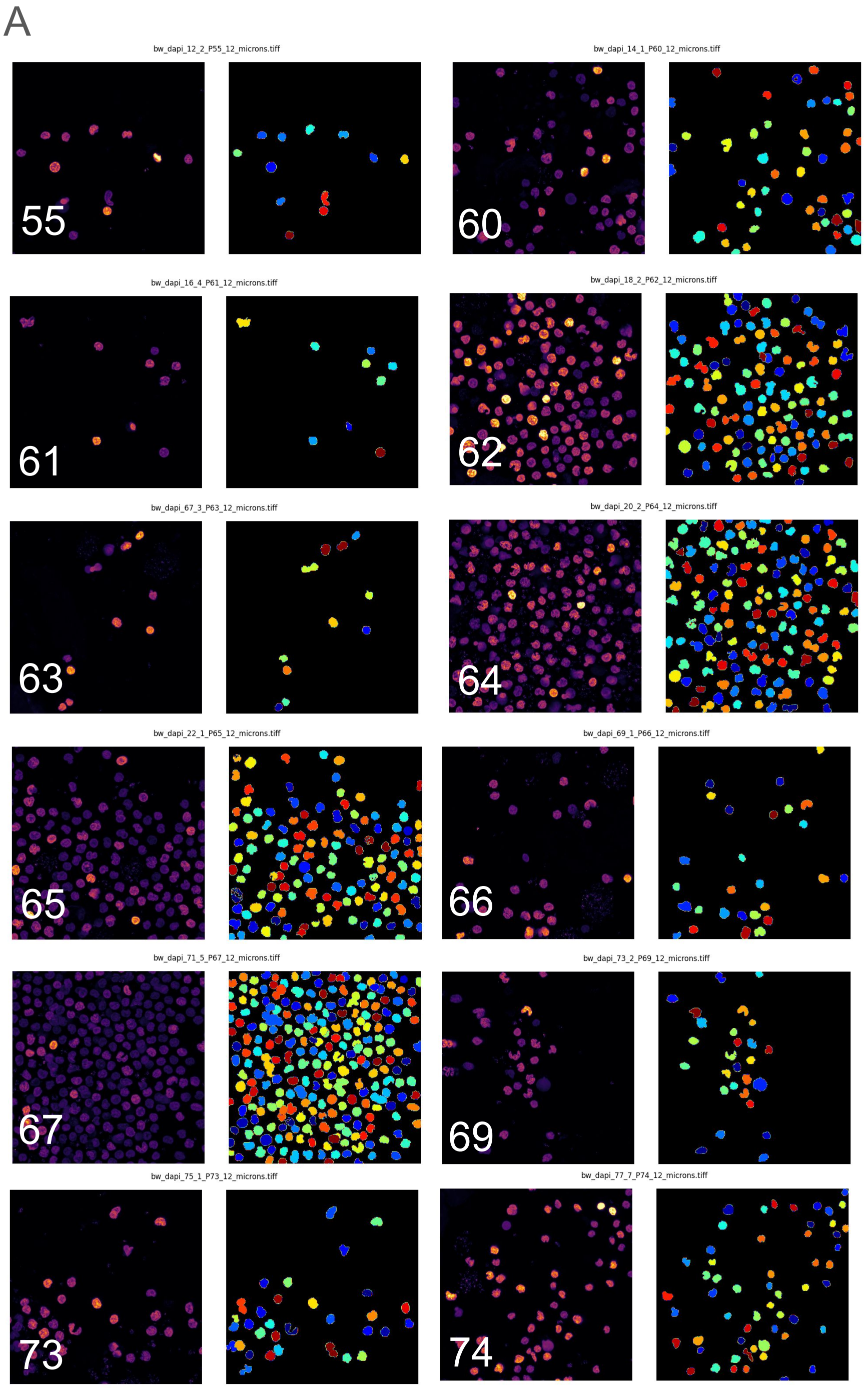

FIG. S3: (A) Sample No. 55 to 74: Representative maximum z projected confocal images stained with Hoechst (left) and corresponding nuclear segmentations (right). The segmentation is colormapped (colormap: jet) and randomly shuffled to enhance visual distinction and clarity. The number annotations to images represent the corresponding Sample No (see Table 1.)

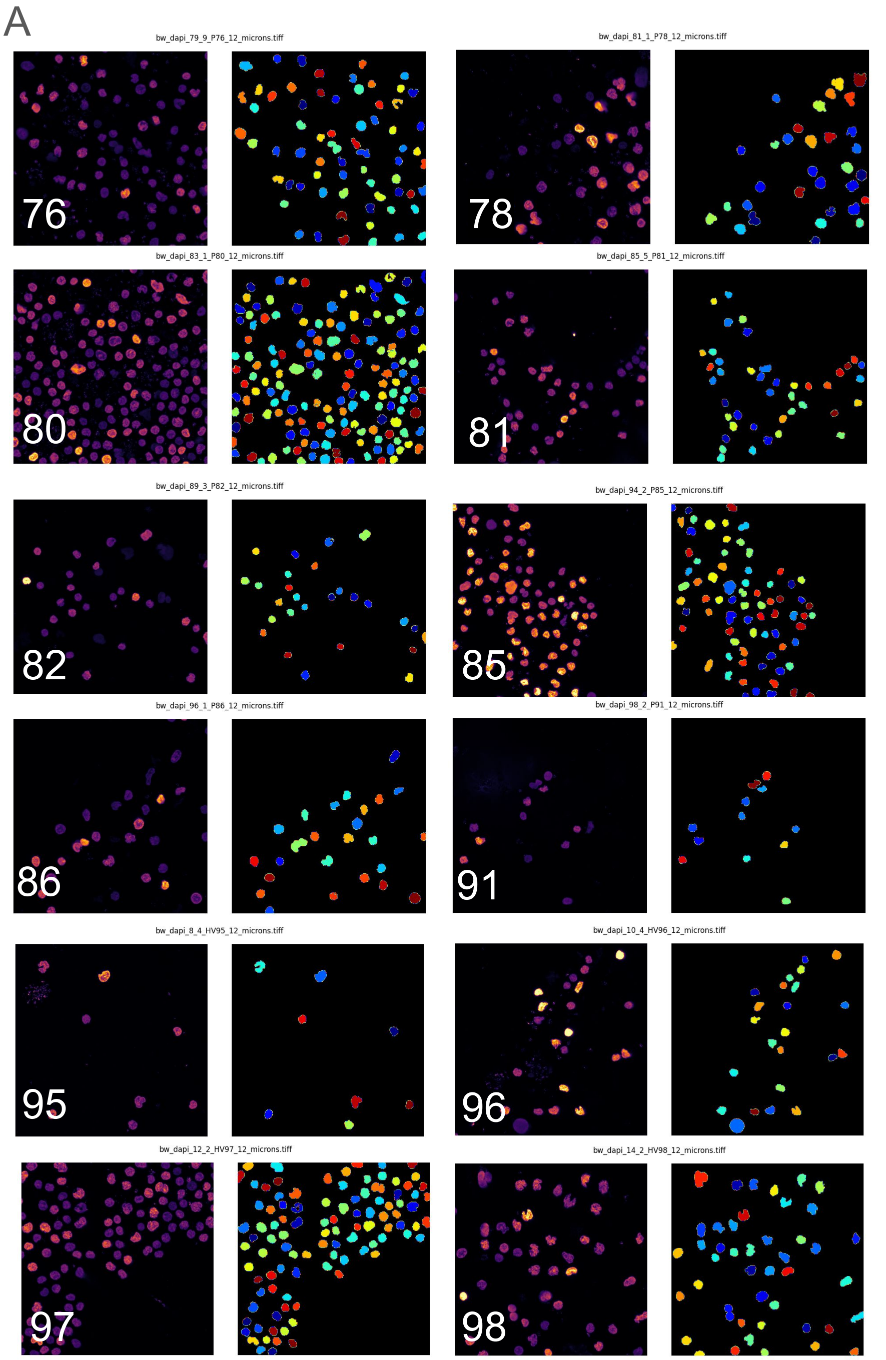

FIG. S4: (A) Sample No. 76 to 98: Representative maximum z projected confocal images stained with Hoechst (left) and corresponding nuclear segmentations (right). The segmentation is colormapped (colormap: jet) and randomly shuffled to enhance visual distinction and clarity. The number annotations to images represent the corresponding Sample No (see Table 1.)

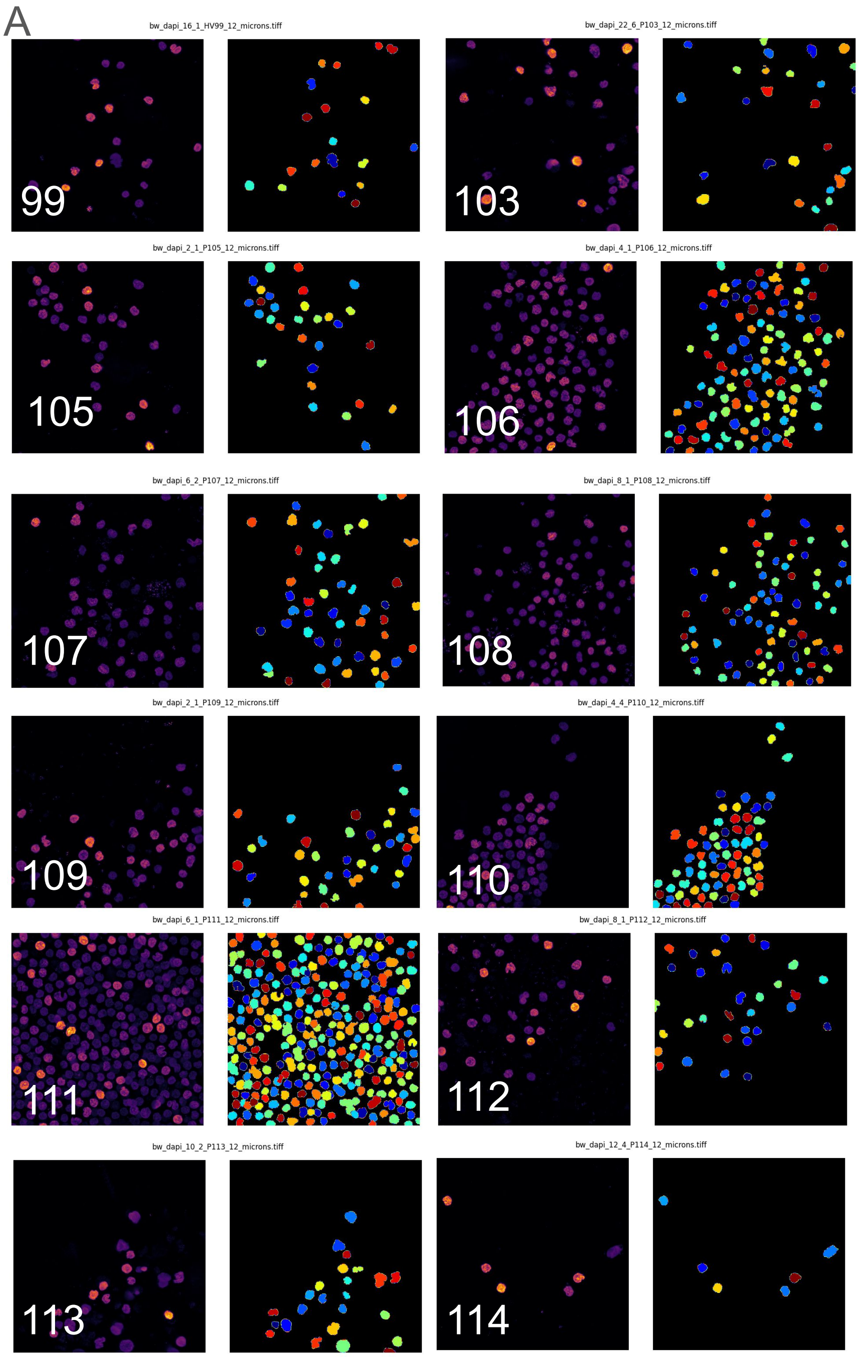

FIG. S5: (A) Sample No. 99 to 114: Representative maximum z projected confocal images stained with Hoechst (left) and corresponding nuclear segmentations (right). The segmentation is colormapped (colormap: jet) and randomly shuffled to enhance visual distinction and clarity. The number annotations to images represent the corresponding Sample No (see Table 1.)

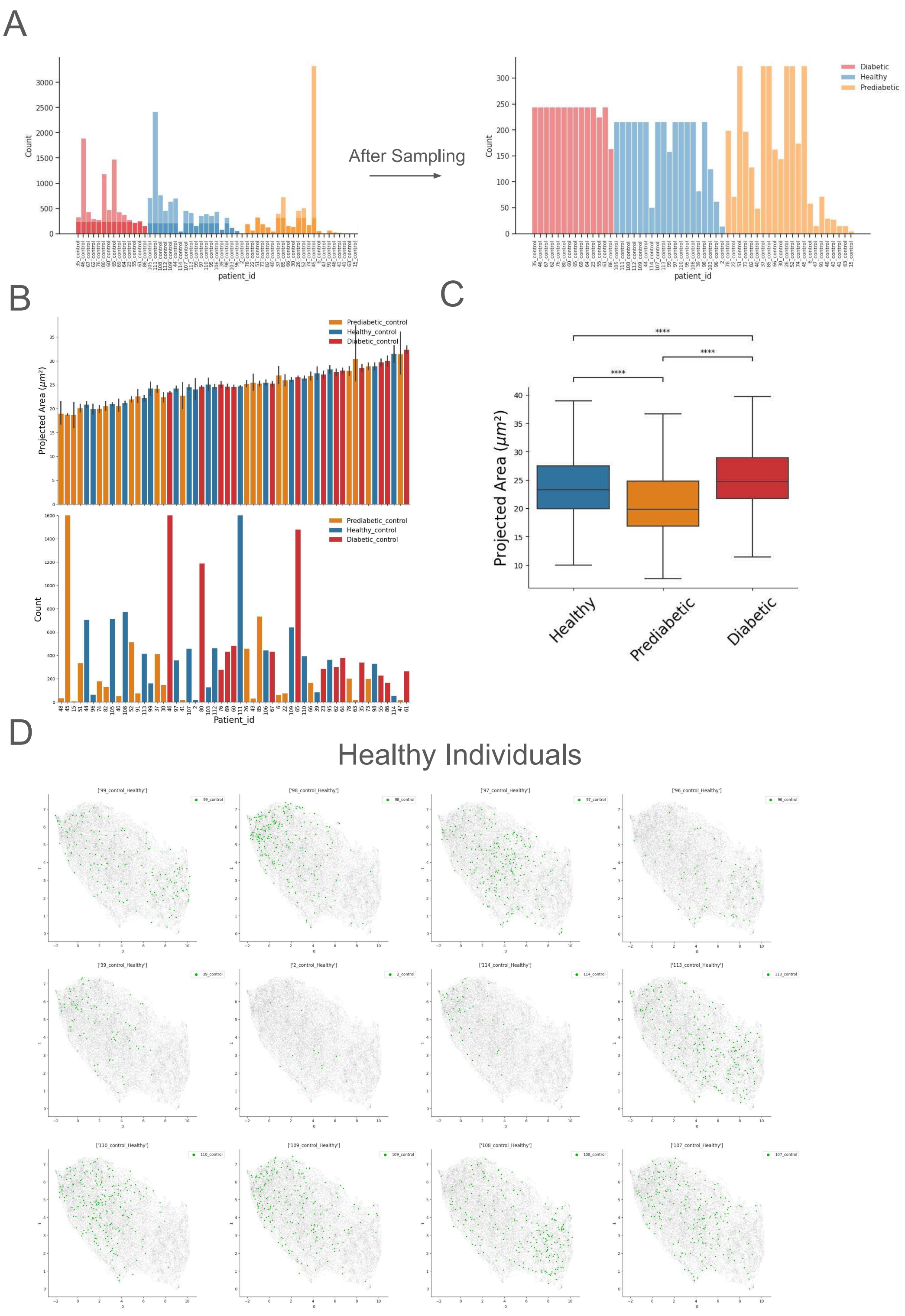

FIG. S6: (A) Bar plot indicates nuclei counts before and after sampling across different patients and different disease stages of T2DM. Balancing is done so that the samples with low nuclear counts are represented without further down-sampling. Samples with high nuclear counts are down-sampled to an intermediate sample count. The total number of nuclei from different disease stages of T2DM is equal (See Methods). (B) Mean nuclear-projected area for different patients. Color coding indicates the disease stage of T2DM in different individuals. Error bar indicating standard error of the mean (s.e.m). Corresponding to the sorted order of median projected area, the bar graph below indicates counts of segmented nuclei for each individual from each of the disease stages of T2DM. (C) Mean z projected nuclear area pooled together for all patients from each disease stage of T2DM. p-value evaluated using the Mann-Whitney U test. (D) VAE-obtained features are dimensionally reduced using UMAP. Annotated green points indicate single nuclei from an individual across various healthy individuals after downsampling.

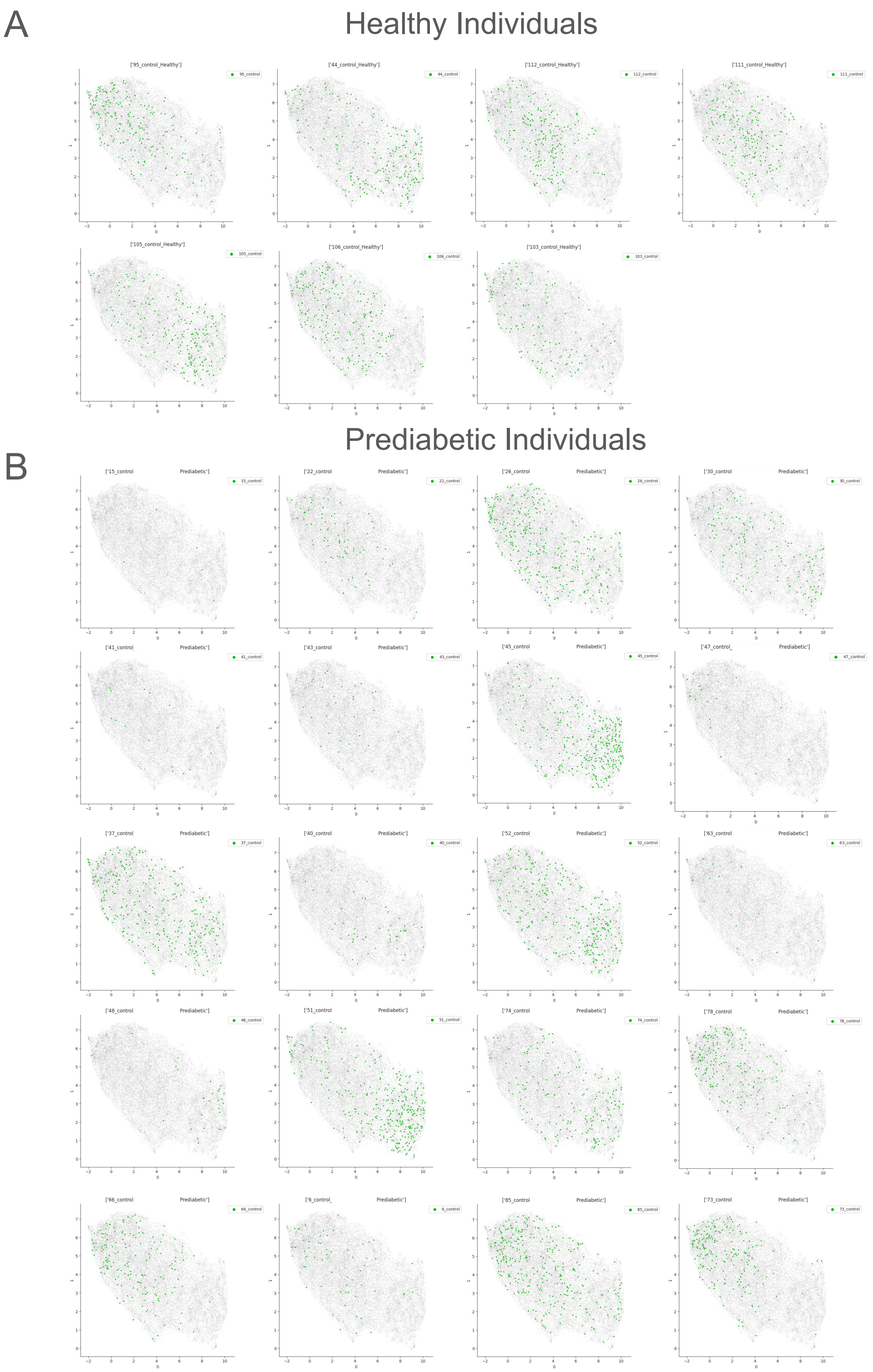

FIG. S7: Annotated green points indicate single nuclei from an individual across various (A)Healthy, and (B) Prediabetic individuals after downsampling.

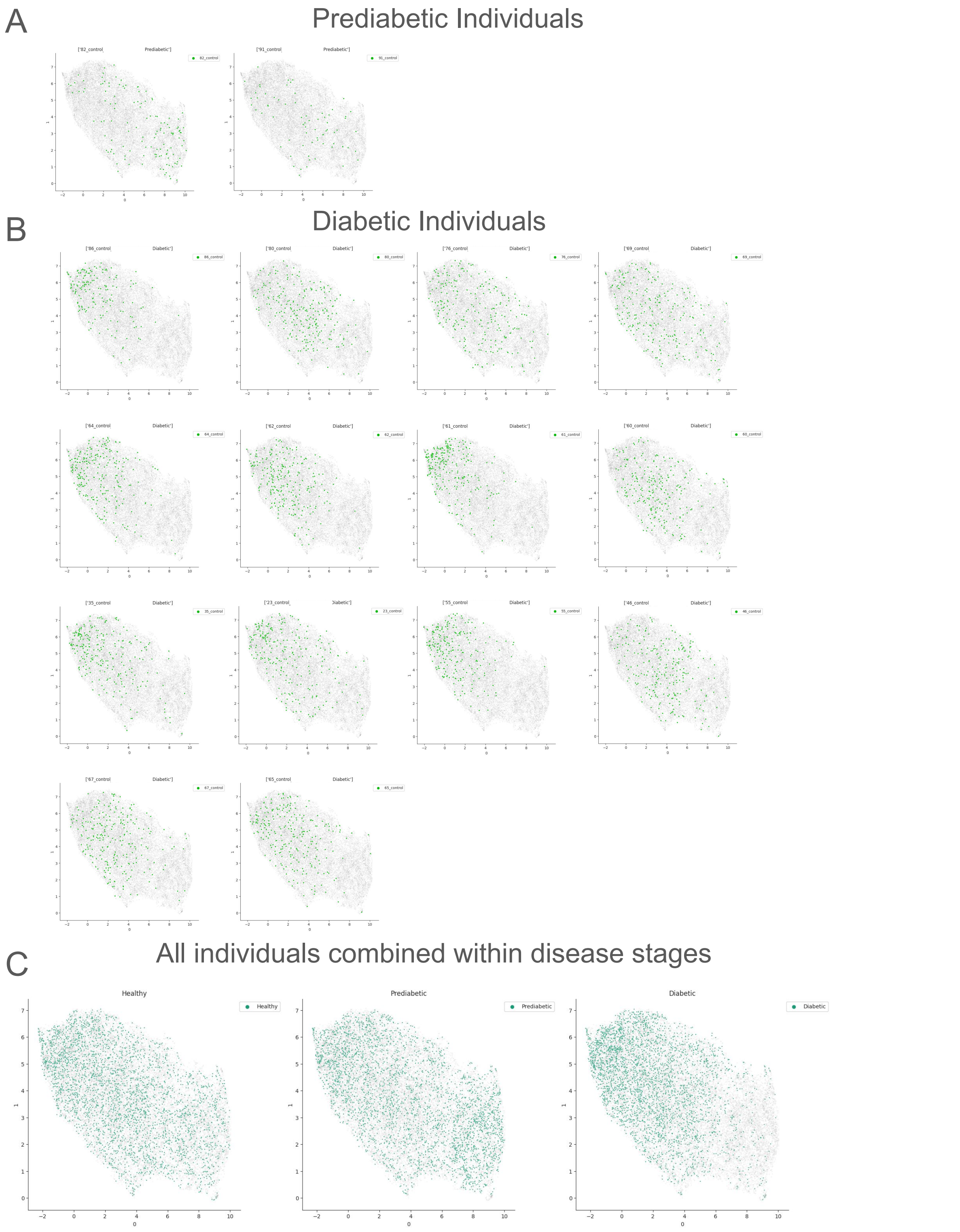

FIG. S8: Annotated green points indicate single nuclei from an individual across various (A)Prediabetic, and (B) Diabetic individuals after downsampling. (C) Annotated green points indicate all the nuclei from different T2DM disease conditions in each scatter plot.

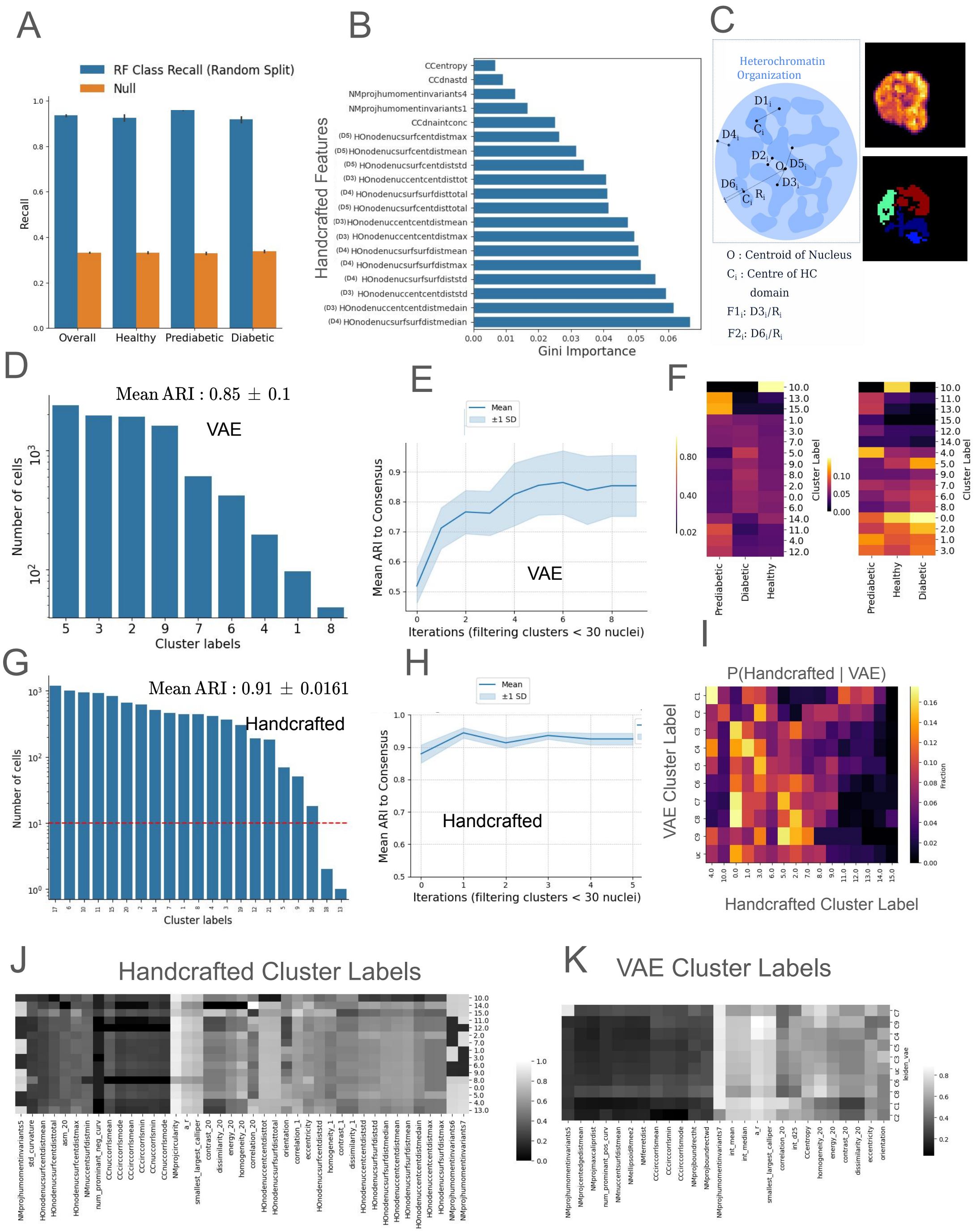

FIG. S9: (A) Mean recall (± s.e.m.) across 5-fold cross-validation for each pathology class (Healthy, Prediabetes, Diabetes), shown alongside a permutation-null baseline obtained by shuffling pathology labels prior to each random train/test split. (B) Random-forest Gini (mean decrease in impurity) feature importances for pathology classification, averaged over random splits. (C) Top handcrafted features capturing heterochromatin organization of nuclei (e.g., texture, intensity, radial distribution), ranked by importance. (D) Sizes of clusters obtained from iterative dense-consensus Leiden clustering in the VAE embedding C_{VAE}; mean Adjusted Rand Index across runs $= 0.85 \pm 0.102$. (E) Cluster-level consensus scores after iteratively removing nuclei from clusters with size < 30. (F) (left) Cluster-specific enrichment profile, $Enrichment_h$ (see Methods), representing the fraction of cells from each T2DM disease stage (column) in a specific cluster (rows). Warmer colors represent a higher fraction of a particular disease stage in a cluster. Row sums to 1. (right) Disease-specific enrichment profile, $Enrichment_d$ (see Methods), representing fraction of cells from different clusters in a disease stage. Colder colors across all columns (for eg, 14 and 15) represent less populous and smaller clusters. Columns sum to 1. (columns), the fraction of nuclei assigned to each VAE cluster label, C_{VAE} (rows); columns sum to 1. (G) Sizes of clusters obtained from iterative dense-consensus Leiden clustering in the Hand-crafted feature embedding C_{HFE}; mean Adjusted Rand Index across runs $=$ 0.91 ± 0.016. (H) Cluster-level consensus scores in C_{HFE} after iteratively removing nuclei from clusters with size < 30. (I) Cross-method association matrix showing, for each VAE-derived cluster labels, C_{VAE} (rows), the fraction of nuclei assigned to each handcrafted feature cluster label, C_{HFE} (columns); rows sum to 1. Differential features among cluster identified from handcrafted features (J) and VAE derived clusters (K).

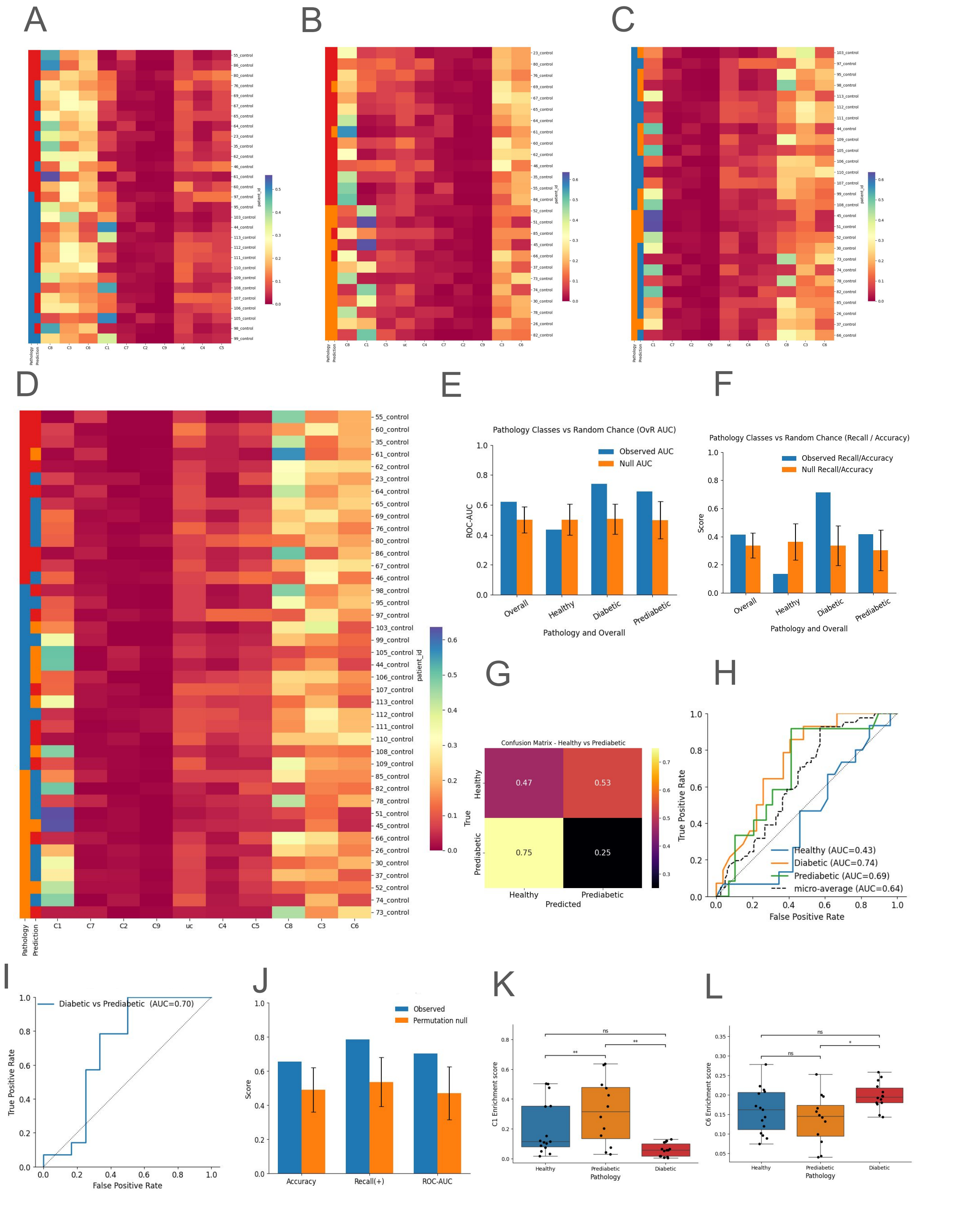

FIG. S10: VAE obtained features and embedding: Heatmap where each row indicates enrichment vector of nuclei from an individual from (A) Healthy and Diabetic, (B) Diabetic and Prediabetic, (C) Healthy and Prediabetic, and (D) All three pathologies across different cluster labels. Each row head annotations indicate the (left) ground truth and (right) predicted pathology. The color map indicates the fraction of nuclei from PBMCs of individual samples in a particular cluster. (E-F) The bar plots indicate the TPR for binary Pathology label predictions averaged over 5-fold cross-validation against randomly shuffled samples. (G) The 2× 2 confusion matrix indicates the TPR for LOOCV classification of healthy vs prediabetic individuals. The ROC curve indicates the probability of TP vs FP for 5-fold cross-validation and prediction of individual pathology if supplied with (H) Diabetes vs prediabetes vs healthy (I) Diabetes vs prediabetes enrichment vectors. (J) Bar plot indicates the recall for class label predictions averaged over 5-fold cross-validation against randomly shuffled samples. (K-L) Box plot representing Enrichment score calculated for Cluster 1 & 6 across different pathologies. Each dot represents an individual.

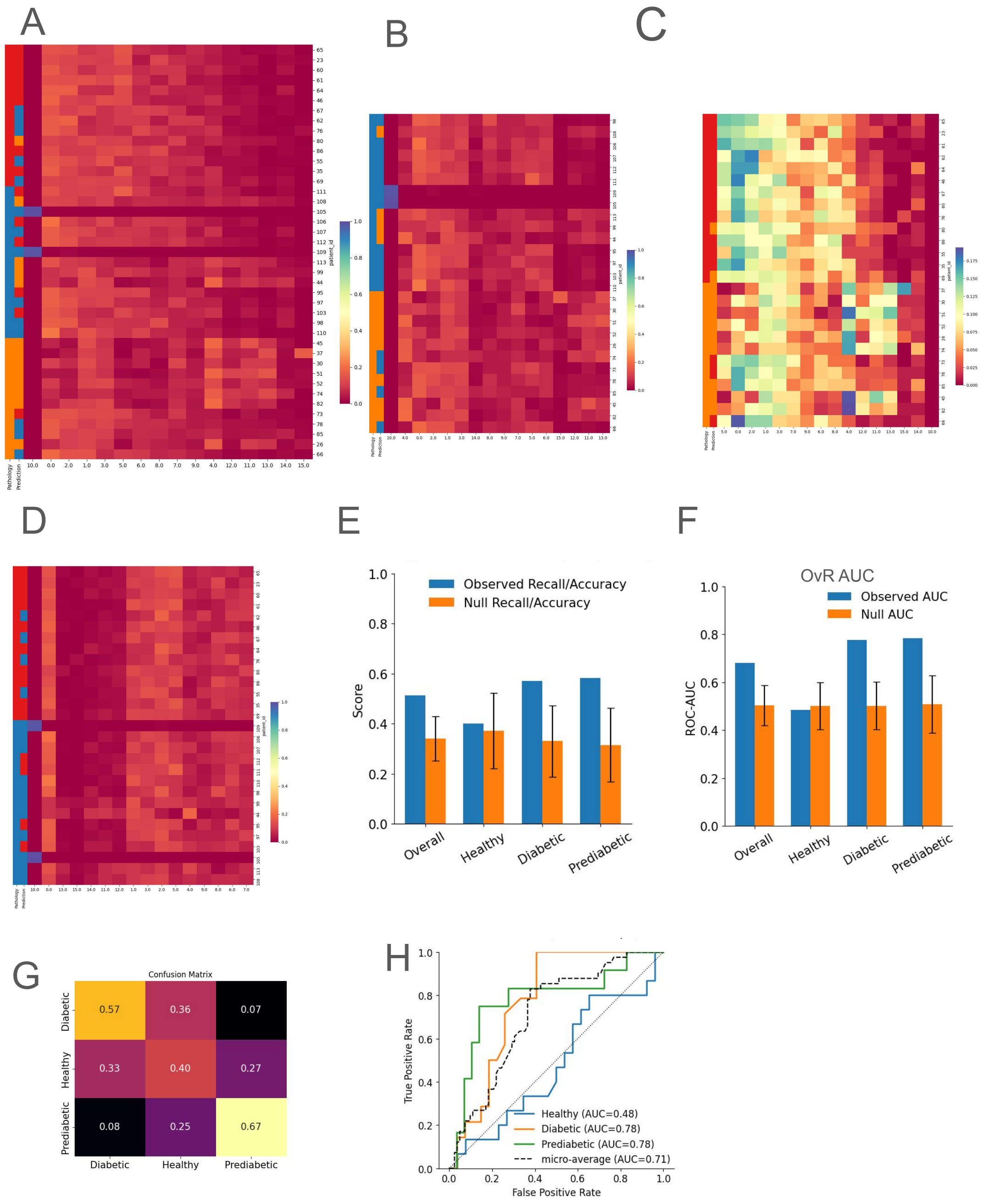

FIG. S11: Handcrafted feature and embedding: Heatmap where each row indicates enrichment vector of nuclei from an individual from (A) All three pathologies (B) Healthy vs Prediabetic,(C) Diabetic vs Prediabetic, and (D) Healthy vs Diabetic across different cluster labels. Each row head annotations indicate the (left) ground truth and (right) predicted pathology. (E) 5-fold cross-validation averaged prediction of Pathology-wise and overall recall against random permutation of Pathology labels as Null (iter = 200). (F) 5-fold cross-validation averaged True positive rate of Pathology binary labels against random label permutation as Null (iter = 200). (G) 3×3 confusion matrix showing LOOCV prediction rates for different pathologies. (H) ROC curve representing false positive probability vs true positive probability of binary class label for different pathologies, and overall true positive probability vs false positive probability.

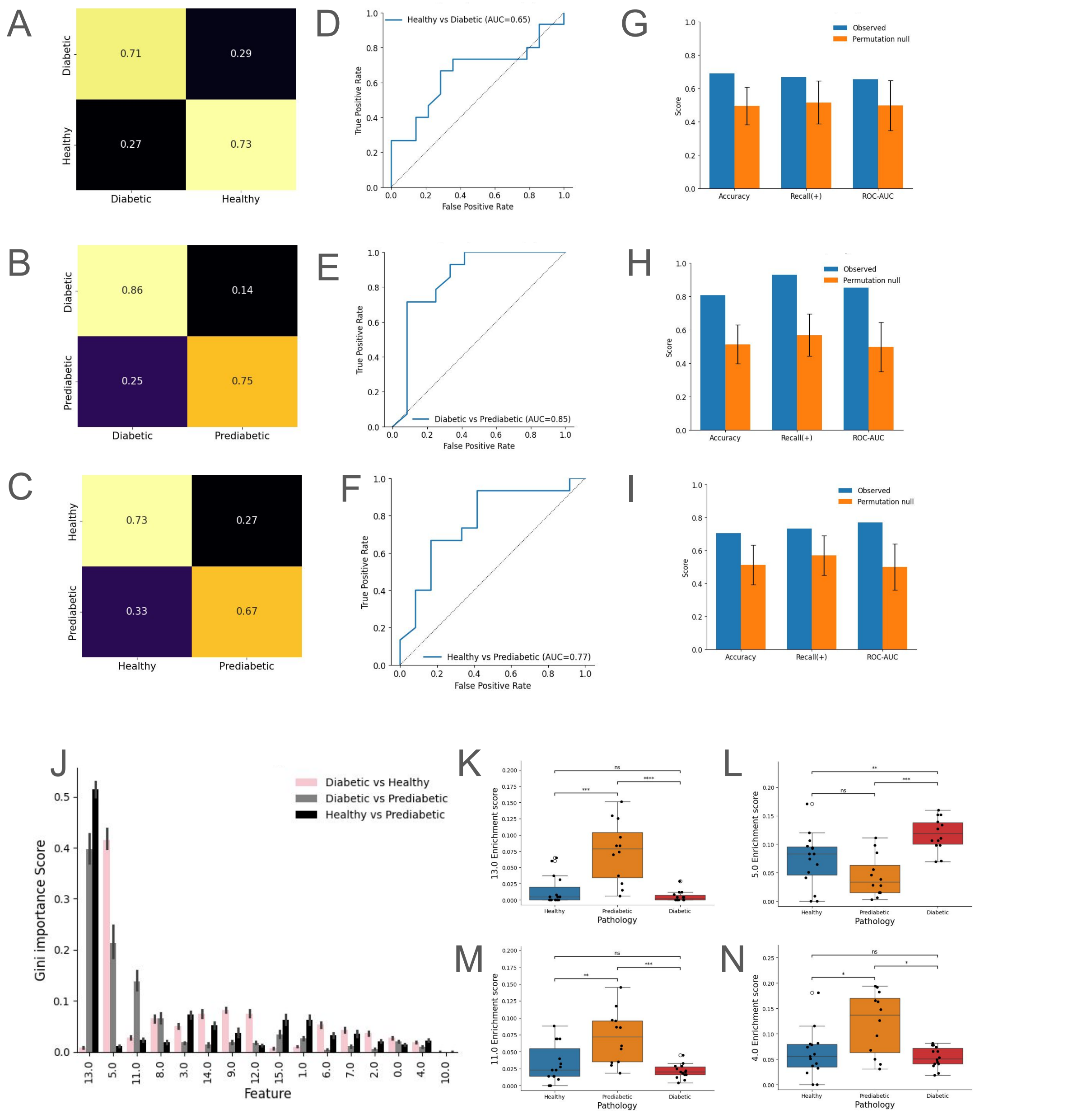

FIG. S12: Confusion matrix showing TPR for LOOCV two-way classification between (A) Healthy vs Diabetic, (B) Prediabetic vs Diabetic, and (C) Healthy vs Prediabetic. ROC curves showing Probability for False Positive prediction vs True positive prediction with 5-fold CV for (D) Healthy vs Diabetic, (E) Diabetic vs Prediabetic, and (F) Diabetic vs Healthy individuals. Bar plot indicates the two-way prediction recall of (G) Healthy vs Prediabetic (H) Prediabetic vs Diabetic (I) Healthy vs Diabetic, averaged over 5-fold cross-validation (blue) against shuffled randomized label permutation (orange). The black annotation on the bar plot marks 1 standard deviation. (J) Cluster GINI importance score averaged over LOOCV 2-way prediction between different pathologies. (K),(L), (M), and (N) Box plot representing Enrichment score calculated for Cluster 13, 5, 11, and 4 of C_{HFE} across different pathologies. Each dot represents an individual.

A

Training

Disease	ID
Healthy	109
Diabetic	35
Diabetic	67
Prediabetic	30
Diabetic	65
Healthy	98
Healthy	110
Healthy	44
Prediabetic	51
Prediabetic	26
Healthy	105
Diabetic	60
Diabetic	76
Healthy	107
Prediabetic	66
Prediabetic	73
Diabetic	80
Prediabetic	45
Diabetic	86
Diabetic	46
Prediabetic	37
Diabetic	61
Diabetic	23
Healthy	95
Prediabetic	82
Healthy	111
Healthy	112
Healthy	108

B

Held out

ID	Ground Truth	All	Dia vs PreDia	PreDia vs Hlty	Hlty vs Dia
55	Diabetic	Diabetic	Diabetic		Diabetic
62	Diabetic	Diabetic	Diabetic		Diabetic
64	Diabetic	Prediabetic	Prediabetic		Diabetic
69	Diabetic	Healthy	Diabetic		Healthy
103	Healthy	Healthy		Healthy	Healthy
106	Healthy	Prediabetic		Healthy	Healthy
113	Healthy	Prediabetic		Healthy	Healthy
97	Healthy	Diabetic		Prediabetic	Diabetic
99	Healthy	Prediabetic		Healthy	Healthy
52	Prediabetic	Prediabetic	Prediabetic	Healthy	
74	Prediabetic	Prediabetic	Prediabetic	Healthy	
78	Prediabetic	Prediabetic	Prediabetic	Healthy	
85	Prediabetic	Healthy	Diabetic	Prediabetic	

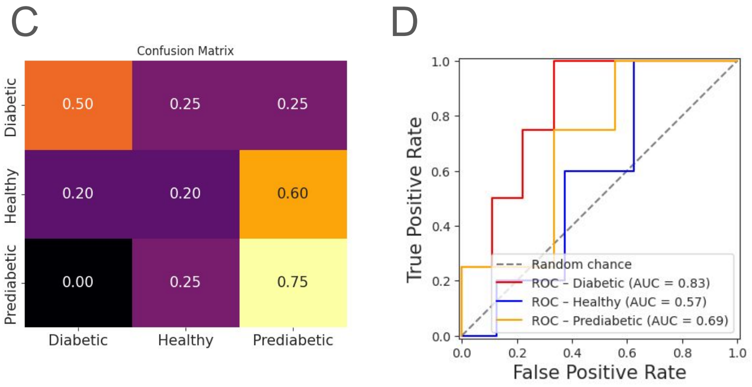

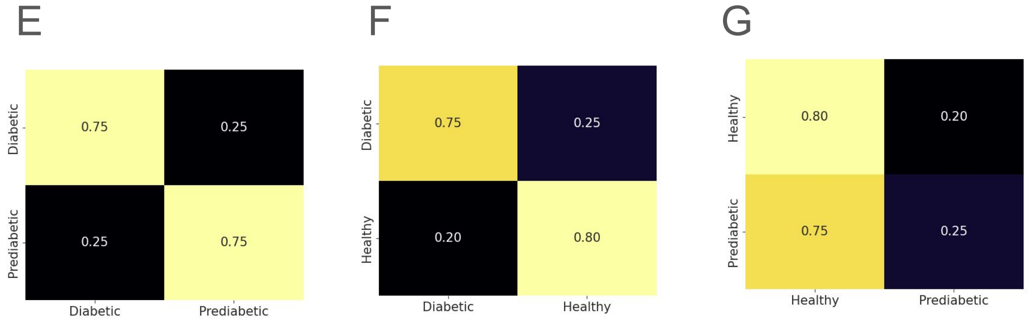

FIG. S13: (A)Individuals sampled for training data, (B) Individuals held out from training data and their pathology prediction, (C) 3×3 Confusion Matrix showing prediction rates across different pathologies. (D) ROC curve representing the false positive rate for the expected true positive rate of the binary class label for different pathologies. 2× Confusion Matrix for classification on (E) Prediabetic vs Diabetic, (F) Healthy vs Diabetic, (G) Healthy vs Prediabetic.

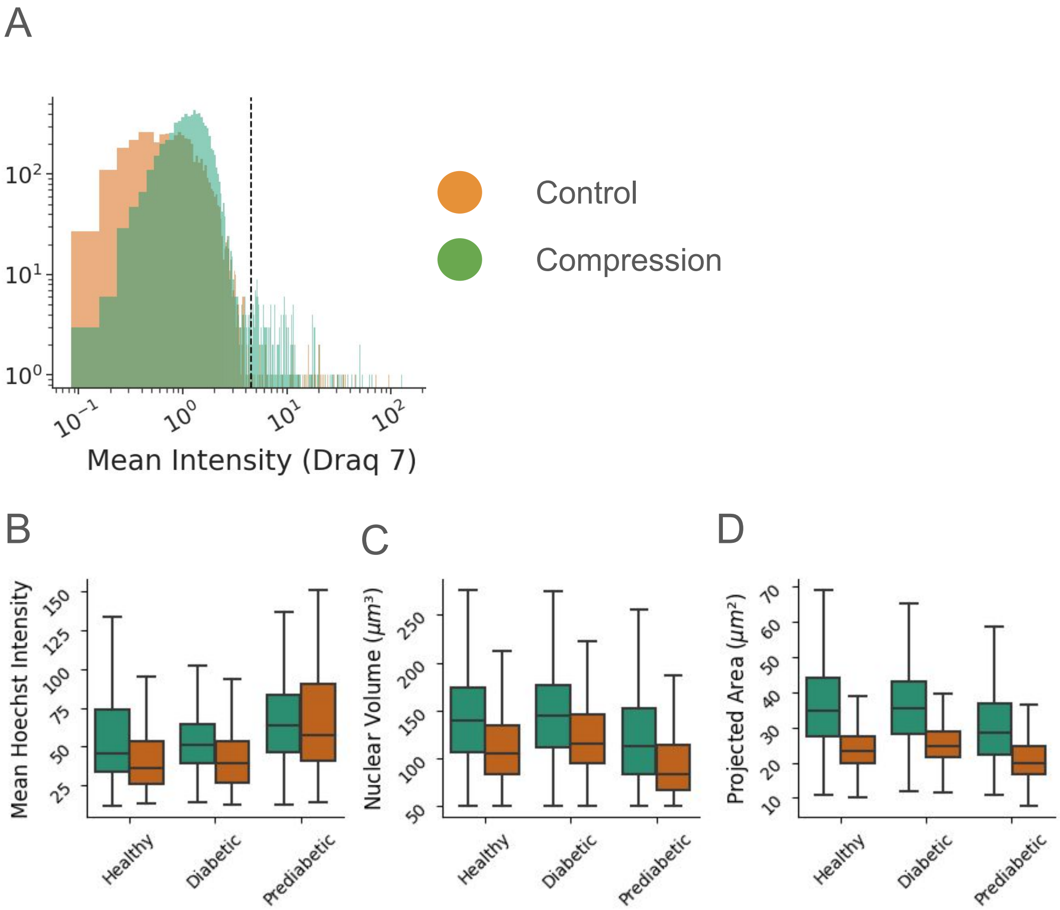

FIG. S14: (A) Log-Log scaled histogram of DRAQ 7 intensity indicating Live-Dead threshold value chosen to filter out the dead nuclei. Box plots indicating the (B) Mean Hoechst intensity (C) Nuclear volume and (D) Projected Area in control and compressed live PBMC nuclei pooled from all the individuals from different stages of T2DM enjoy high statistical significance because of the high nuclei count. Color coding indicates control or compressed condition.

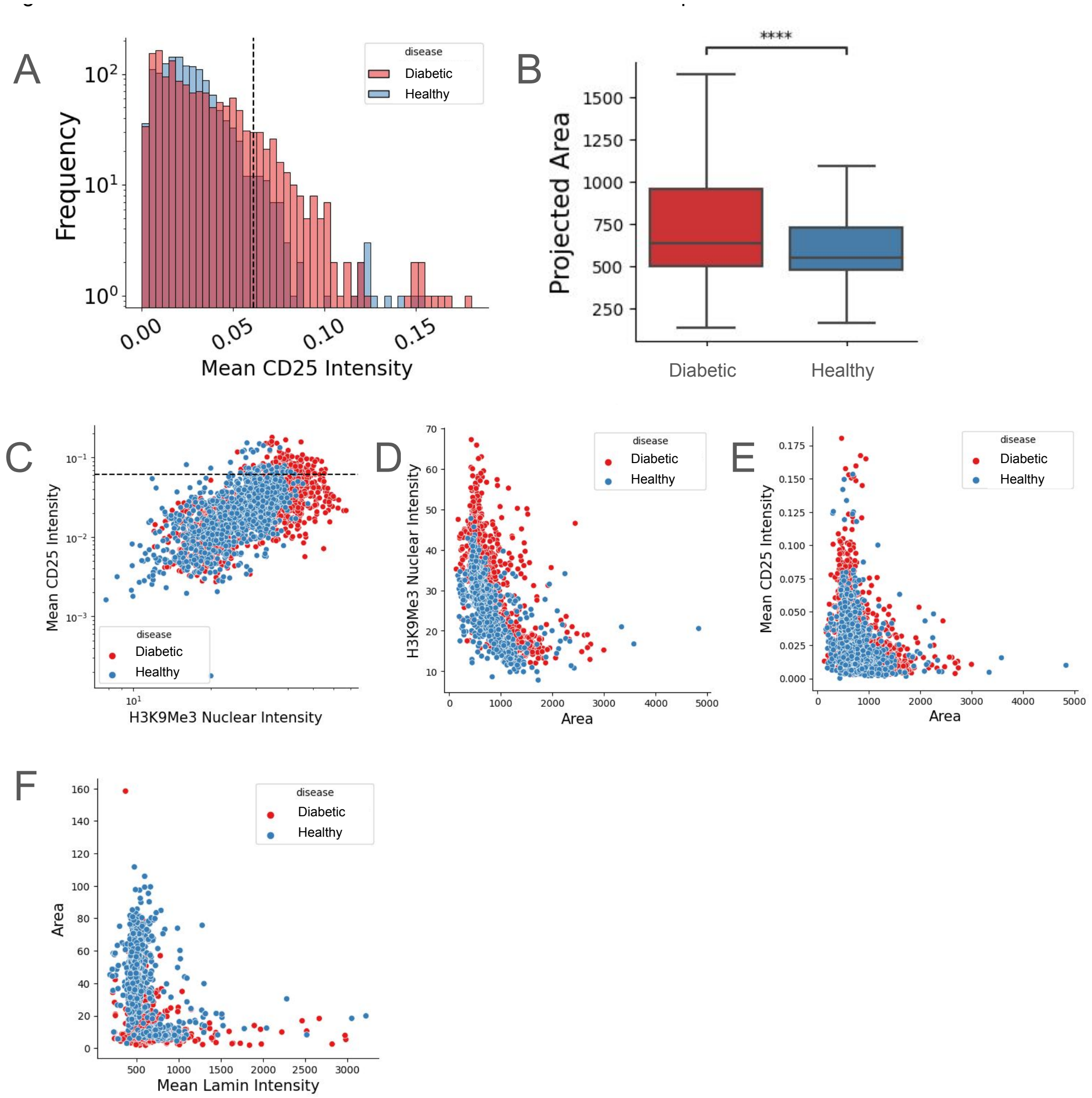

FIG. S15: (A) Log-Log scaled histogram of CD25 intensity indicating CD25+ threshold value chosen to analyse activated nuclei. (B) Mean projected area of nuclei of PBMCs in diabetic and healthy individuals after immunostaining protocols. (C) The scatter plot of H3K9Me3 vs CD25 immunofluorescence intensity of individual nuclei from healthy and diabetic individuals. Projected area vs (D) H3K9Me3 (E) CD25 (F) Lamin immunofluorescence intensity from Healthy and Diabetic individuals.

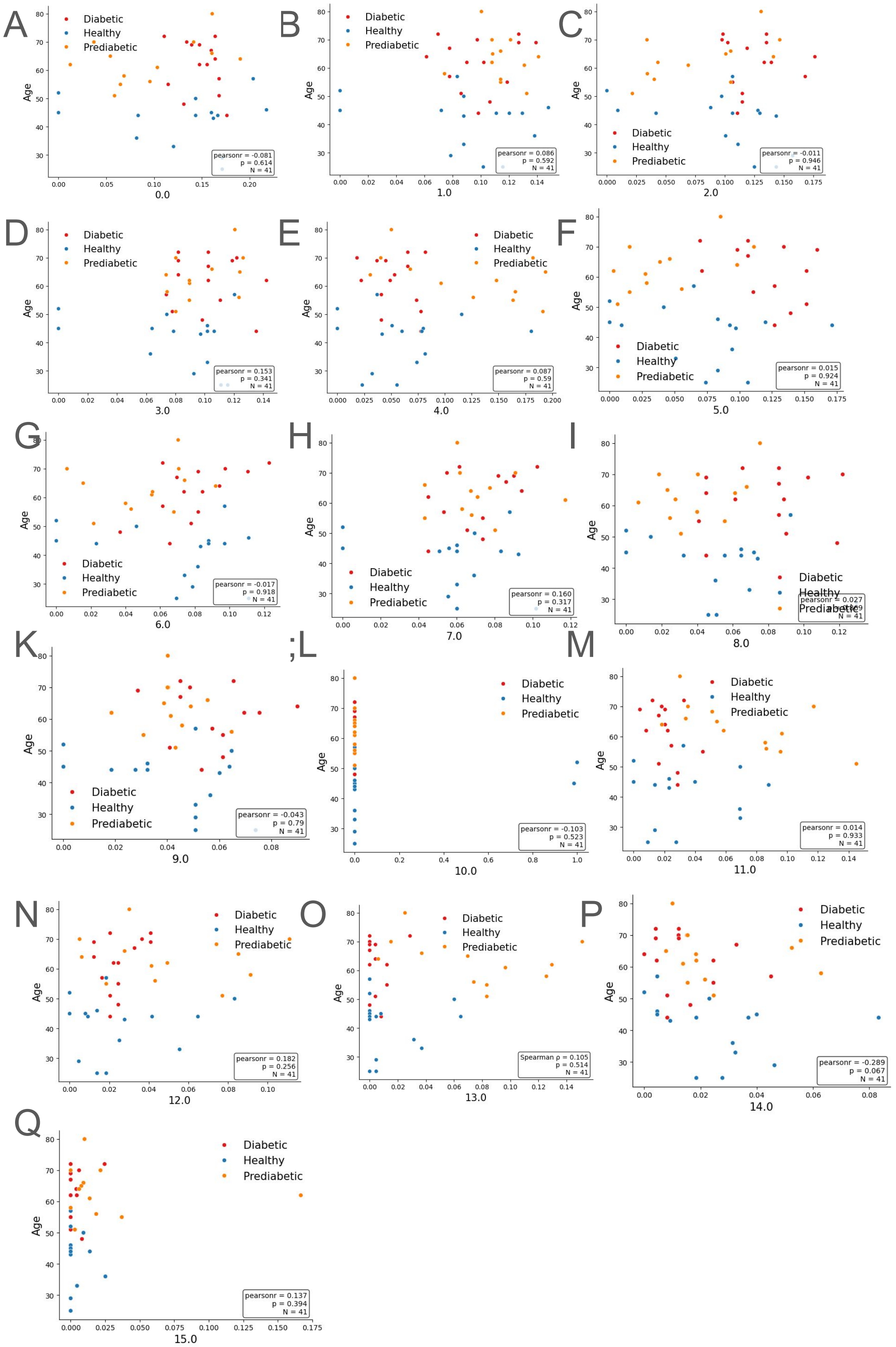

FIG. S16: A-Q Scatter plot annotated with Pearson R and corresponding p Value with Age, color coded for individuals with different pathologies.

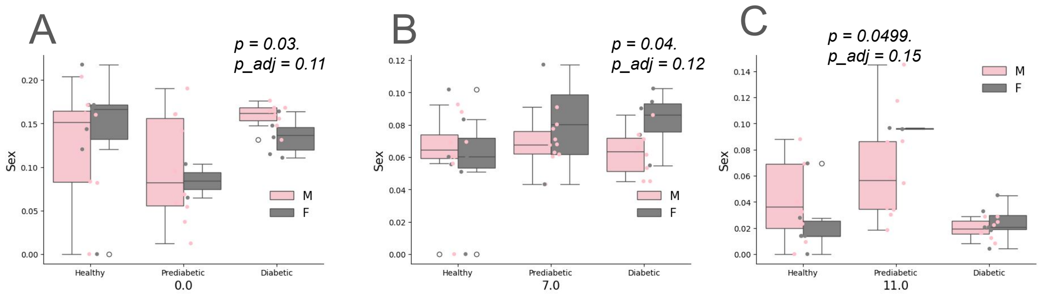

FIG. S17: A-C Box plot showing association with Sex with individuals with different pathologies. p Value (welch) unless stated is non-significant, p Adj (Benjamini-Hochberg).

REFERENCES

[1] CM Bennett, M Guo, and SC Dharmage. “HbA1c as a screening tool for detection of type 2 diabetes: a systematic review”. In: *Diabetic medicine* 24.4 (2007), pp. 333–343.

[2] James B Meigs. *Multiple biomarker prediction of type 2 diabetes.* 2009.

[3] Iren D Hjellestad et al. “HbA1c versus oral glucose tolerance test as a method to diagnose diabetes mellitus in vascular surgery patients”. In: *Cardiovascular diabetology* 12.1 (2013), p. 79.

[4] Ge Li et al. “Evaluation of ADA HbA1c criteria in the diagnosis of pre-diabetes and diabetes in a population of Chinese adolescents and young adults at high risk for diabetes: a cross-sectional study”. In: *BMJ open* 8.8 (2018), e020665.

[5] Aruna D Pradhan et al. “C-reactive protein, interleukin 6, and risk of developing type 2 diabetes mellitus”. In: *jama* 286.3 (2001), pp. 327–334.

[6] Shaivya Gupta, U Jain, N Chauhan, et al. “Laboratory diagnosis of HbA1c: a review”. In: *J Nanomed Res* 5.4 (2017), p. 00120.

[7] Rachael Hunter. “Cost-effectiveness of point-of-care C-reactive protein tests for respiratory tract infection in primary care in England”. In: *Advances in therapy* 32.1 (2015), pp. 69–85.

[8] FindLabtest.com. Accessed: 2025-09-04. 2025. URL: `https://www.findlabtest.com/lab-test/fertility-test/interleukin-6-il-6-serum-quest-34473#:~:text=Interleukin,00`.

[9] https://www.ufluidix.com/pricing/. *uFluidix Pricing Information.* Accessed: 2025-09-04. 2025. URL: `https://www.ufluidix.com/pricing/`.
